# In some cases more complicated approaches to refinement of macromolecular structures are not necessary

**DOI:** 10.1107/S2052252524005803

**Published:** 2024-06-27

**Authors:** Zbigniew Dauter, Alexander Wlodawer

**Affiliations:** a Center for Structural Biology, Center for Cancer Research National Cancer Institute Frederick Maryland USA; University of Auckland, New Zealand

**Keywords:** protein structure refinement, *Amber*, molecular dynamics, maximum-likelihood

## Abstract

The manuscript ‘Modeling a unit cell: crystallographic refinement procedure using the biomolecular MD simulation platform *Amber*’ presents a novel protein structure refinement method claimed to offer improvements over traditional techniques like *Refmac5* and *Phenix*. Our re-evaluation suggests that while the new method provides improvements, traditional methods achieve comparable results with less computational effort.

In the manuscript published in *IUCrJ* under the title ‘Modeling a unit cell: crystallographic refinement procedure using the biomolecular MD simulation platform *Amber*’ (Mikhailovskii *et al.*, 2022 ▸), the authors described the results of the refinement of 84 protein structures using a novel approach that utilizes molecular dynamics and the maximum-likelihood potential that encodes the structure-factor based restraints. They claimed the procedure was superior to traditional approaches used for refinement of macromolecular structures, exemplified by programs such as *Refmac5* (Murshudov *et al.*, 2011 ▸) or *Phenix* (Adams *et al.*, 2010 ▸). In our opinion, the only example for which the re-refined structure was deposited in the Protein Data Bank (PDB) does not provide convincing proof for the superiority of this refinement method.

The structure chosen as an example represented Type III antifreeze protein isoform HPLC12 (PDB entry 2msi; DeLuca *et al.*, 1998 ▸). The structure was originally refined at 1.9 Å resolution, but it appears that the refinement was not finalized. In particular, the deposited coordinates lacked any solvent that should have been visible at this resolution, thus despite good validation statistics (Table 1 ▸), that structure has to be considered of uncertain quality.

In the procedure used by Mikhailovskii *et al.* (2022 ▸), the original unit cell that contained a single protein molecule in the asymmetric unit in the space group *P*2_1_2_1_2_1_ was expanded to *P*1 with identical unit-cell parameters, but containing four independent molecules. Thus, the number of refinable parameters was significantly increased, though the amount of available diffraction data remained the same. Their re-refined structure consisted of 1952 protein atoms and 155 water molecules (Table 1 ▸). The resulting model (PDB entry 7q3v) corrected obvious mistracing at the N-terminus and added missing solvent. As described by the authors, this was accomplished after very extensive calculations that could only be done on a computer equipped with GPUs.

To put this effort into perspective, we re-refined the structure 2msi with *Refmac5*, after manually correcting the erroneous tracing of the N-terminus and several improper side-chain rotamers with the program *Coot* (Emsley *et al.*, 2010 ▸). Waters were added automatically in *Coot* on the basis of 3.5σ peaks in the *F*_o_ − *F*_c_ electron density map. Statistics of the resulting structure (PDB entry 9cbe), some obtained with *MolProbity* (Chen *et al.*, 2010 ▸), are also shown in Table 1 ▸. The process took less than an hour of human time and negligible computing time on a standard PC.

In principle, the more adjusted parameters, the better the agreement between the observed and calculated functions. However, if more parameters do not lead to better agreement, the strict Ockham razor should be mercilessly applied to avoid unnecessary complication of the problem. It is clear that, at least in this case, the complicated procedure involving molecular dynamics did not work any better than manual refitting and standard crystallographic refinement. Although the procedure utilized by Mikhailovskii *et al.* (2022 ▸) might be potentially very useful in some specific cases, the sole example out of the 84 that were deposited in the PDB was maybe not an optimal selection. A more difficult or complicated case might have provided a clearer indication of the usefulness of this novel structure refinement procedure.

## Figures and Tables

**Table 1:** Selected statistics of the three models of Type III antifreeze protein isoform HPLC12

	Original structure (PDB entry 2msi)	*Amber*-refined (PDB entry 7q3v)	*Refmac5*-refined (PDB entry 9cbe)
Protein atoms (non-hydrogen)	485	1952	495
Solvent atoms	0	155	39
*R*/*R*_free_	0.193/0.261	0.160/0.194	0.152/0.189
Clashscore	3.99 (98th percentile)	4.45 (97th percentile)	1.94 (100th percentile)
*MolProbity* score	1.61 (92nd percentile)	1.64 (90th percentile)	1.37 (98th percentile)
Bad bonds	0	0	0
Bad angles	8	16	1
